# Genome-Wide Analysis of Differentially Expressed microRNA in *Bombyx mori* Infected with Nucleopolyhedrosis Virus

**DOI:** 10.1371/journal.pone.0165865

**Published:** 2016-11-02

**Authors:** Ping Wu, Xiaoxu Jiang, Xijie Guo, Long Li, Tao Chen

**Affiliations:** 1 Sericultural Research Institute, Jiangsu University of Science and Technology, Zhenjiang Jiangsu, China; 2 Quality Inspection Center for Sericulture Products, Ministry of Agriculture, Zhenjiang Jiangsu, China; 3 School of Biology, Jiangsu University of Science and Technology, Zhenjiang Jiangsu, China; Institute of Plant Physiology and Ecology Shanghai Institutes for Biological Sciences, CHINA

## Abstract

*Bombyx mori* nucleopolyhedrosis virus (BmNPV) is a major pathogen that threatens the growth and sustainability of the sericulture industry. Since microRNAs (miRNAs) have been shown to play important roles in host-pathogen interactions, in this study we investigated the effects of BmNPV infection on silkworm microRNAs expression profile. To achieve this, we constructed and deep-sequenced two small RNA libraries generated from BmNPV infected and un-infected larvae. The results revealed that 38 silkworm miRNAs were differentially expressed after BmNPV infection. Based on the GO analysis, their predicted target genes were found to be involved in diverse functions such as binding, catalytic, virion and immune response to stimulus suggesting their potential roles in host-virus interactions. Using the dual-luciferase reporter assay, we confirmed that Bmo-miR-277-5p, up-regulated in BmNPV-infected larvae, targeted the *B*. *mori* DNA cytosine-5 methyltransferase (Dnmt2) gene which may play potential role in silkworm-BmNPV interaction. These results provide new insights into exploring the interaction mechanism between silkworm and BmNPV.

## Introduction

The mulberry silkworm, *Bombyx mori*, serves as a model organism to study insect development and immunity. Silkworm is particularly susceptible to the *B*. *mori* nucleopolyhedrosis virus (BmNPV), which is one of the major pathogens that remain a big challenge to the sericulture industry. BmNPV is a circular double-stranded DNA (dsDNA) virus and belongs to the family *Baculoviridae* [1]. DNA replication and gene expression in this virus follow a cascade reaction pattern with successful infection depending on stage specific expression of BmNPV genes [2]. BmNPV infection causes high larval mortality and significantly damages to silk production [3,4].

MicroRNAs (miRNAs) are 18–25 nucleotides (nts) small non-coding RNAs processed from the stem-loop precursor termed as the pre-miRNA. Growing evidence reveals that miRNAs are key players in regulating immunity and host-pathogen interactions [5–8]. Several studies have reported changes in the expression profile of host cellular miRNAs after a viral infection [9–12]. Previous studies on BmNPV investigated how virus-encoded miRNA regulates BmNPV replication by interacting with BmNPV genes [13–14]. Thus far, only four miRNAs have been reported to target the BmNPV ORFs encoding cathepsin, chitinase, DNA binding protein and vp80 [15]. Recent emerging studies reveal that not only viral miRNAs but also the host cellular miRNAs play critical roles in viral infection. For example, bmo-miR-278-3p positively regulates the mRNA expression of *B*. *mori* cytoplasmic polyhedrosis virus (BmCPV) by interacting with IBP2 (Insulin-related peptide binding protein 2) in silkworm larvae [16]. Similarly, Hz-miR-24 regulates DdRP (DNA dependent RNA polymerase) expression in *Heliothis virescens AV* (HvAV-3e) [17]. Besides, Bmo-miR-8 was identified as an anti-viral miRNA, which is suppressed by BmNPV following infection or transfection of abmnpv-miR-1 into the host cell [13]. MiR-32 inhibits the accumulation of primate foamy virus type 1[18]. Thus, the expression level of host miRNAs may be induced or inhibited after viral infection and may help their replication by targeting viral or host genes.

In this study, we focused on the host cellular miRNAs at the genome-wide level, which revealed 38 differentially expressed silkworm miRNAs involved in BmNPV infection based on deep sequencing. Among these, 9 miRNAs were validated to be differentially expressed using the stem-loop qRT-PCR. We found that bmo-miR-277-5p targeted the *B*. *mori* DNA cytosine-5 methyltransferase (Dnmt2) as shown by dual-luciferase reporter assay. These results may provide new clue to explore interaction between silkworm and BmNPV.

## Materials and Methods

### Silkworm strain and virus inoculation

The P50 silkworm strain was reared at room temperature and under a photoperiod of 12h light and 12h dark until the fourth molt. For viral inoculation, BmNPV viral stock was suspended in distilled water at a concentration of 10^9^ polyhedra/ml. Mulberry leaves treated with BmNPV were fed to 20 newly exuviated fourth instar larvae. The dose of infection was about 1 × 10^6^ polyhedra per larva. The control un-infected larvae were fed on mulberry leaves treated with 0.9% NaC1 solution.

### RNA isolation

Each of ten BmNPV-infected and un-infected control larvae were collected to extract RNA using the Trizol reagent (Invitrogen, USA) according to the manufacturer’s protocol. Following quality inspection, the total RNAs were stored in -80°C for further experiments.

### Small RNA library construction, Solexa sequencing and data analysis

Two small RNA libraries were prepared from un-infected silkworm larvae and those infected with BmNPV 72h post-infection. Each library pooled equal amount of total RNA from 10 infected or un-infected larvae. Small RNA library construction and Solexa sequencing were performed by CapitalBio Corporation (Beijing, China). The detailed workflow and data analysis were as described in our previous study [19]. The screened unique reads were mapped onto miRBASE (Release 21.0) (http://www.mirbase.org/), and perfectly matched sequences were defined as known miRNAs.

### Prediction of novel miRNAs

Low abundance reads (counts < 2) and non-coding RNA including rRNA, tRNA and snoRNA were removed to identify novel miRNAs. The candidate unique reads were searched against the SilkDB (http://silkworm.genomics.org.cn/). Secondary structure was analyzed using Einverted of Emboss [20] and RNA fold program [21] based on extracted 150 nts upstream and downstream of candidate unique reads.

### Prediction of target genes and Gene ontology analysis

Three popular target-predicting software, PITA, Targetscan and miRanda [22–24] were used to predict potential target genes by aligning candidate miRNAs against 3’-UTR sequences, which were extracted from the downstream 1000 nt of silkworm mRNA CDS. Automatic GO analysis of Molecule Annotation System (MAS3.0) was used to analyze the function of predict target genes for the differentially expressed miRNA in BmNPV infected larvae.

### RT-PCR analysis

A total of 1μg RNA was reverse transcribed in a 20μl reaction system using the Prime Script™ RT Reagent Kit (TaKaRa). Reverse transcript products were generated using specific stem-loop primers designed based on the method described by Chen [25] under the following condition: denaturation for 2 min at 94°C, 30 cycles of 94°C for 40 s, 58°C for 40 s and 72°C for 40 s, and then 72°C for 5 min. The *B*. *mori* U6 snRNA gene was amplified as a positive control. The PCR products were analyzed on a 2% agarose gel.

### Differentially expressed miRNAs

The miRNA count was normalized to TPM (number of transcripts per million clean tags) to compensate for variable numbers of tags generated for each sample. MiRNAs with a P value < = 0.05 and log_2_FC>1 or log_2_FC<-1 were identified as significantly differentially expressed in BmNPV infected larvae compared to un-infected larvae.

### Quantitative real-time PCR

Real-time PCR reactions were carried out on an ABI 7300 machine (Applied Biosystems, USA) and performed in a 20 μl reaction volume according to the manufacturer's instructions in the SYBR Premix Ex Taq™ kit (Takara, China) with thermal cycling parameters at 95°C for 30 s followed by 40 cycles of 95°C for 5 s, and 60°C for 31 s. Following amplification, melting curves were constructed. The silkworm U6 snRNA were used as a reference gene for normalization respectively. Three biological replicates with three technical replicates were analyzed. A relative quantitative method (2^-△△Ct^) was used to assess differences in gene expression levels. Primer sequences used are shown in S4 Table.

### Dual-luciferase reporter assay

*Bombyx mori* Dnmt2 3’UTR containing the potential target-binding sequences for miR-277-5p and mutated Dnmt2 3’UTR were synthesized by GenePharma (Shanghai, China). Dnmt2 3’UTR as well as mutated 3’UTR were cloned into the PUC57 vector and verified by sequencing. Correct Dnmt2 3’UTR and mutated 3’UTR were cloned downstream of firefly luciferase in the PmirGLO vector (Promega) using *Nhe*I and *Sal*I restriction sites to create PmirGLO-Dnmt2 and PmirGLO-Dnmt2-mut constructs. MiR-277-5p mimics and negative controls synthesized by GenePharma (Shanghai, China) with PmirGLO, PmirGLO-Dnmt2 and PmirGLO-Dnmt2-mut constructs were co-transfected into 293 cells using lipofectamine2000 reagent (Invitrogen) according to the manufacturer's protocol. Cells were harvested 48h after transfection and analyzed for Firefly and Renilla luciferase activities using the Dual-Luciferase Reporter Assay System (Promega). Activities were normalized to Renilla luciferase. Three independent experiments were performed in triplicate.

## Results

### Confirmation of BmNPV infection

Under the dose of 1× 10^6^ polyhedra per larva, all the infected silkworm larvae showed typical symptom with tense and milk white body wall. Some larvae appeared bamboo-like structure due to the apophysis of intersegmental membrane. BmNPV infection were further validated with appearance of a lot of polyhedra under microscope.

### Overview of two sequenced small RNA libraries

**Fig 1:**
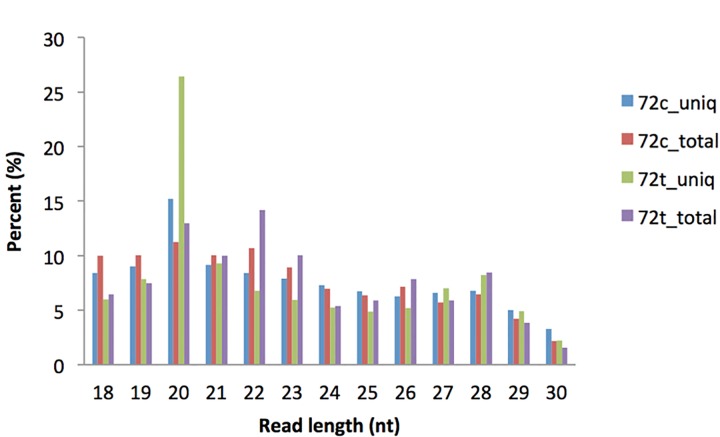
Size distribution of small RNA in two small RNA libraries. 72c_uniq: unique clean reads in 72h un-infected small RNA library. 72c_total: total clean reads in 72h un-infected small RNA library. 72t_uniq: 72h post-BmNPV-inoculated small RNA library. 72t_total: total clean reads in 72h post-BmNPV-inoculated small RNA library.

Two small RNA libraries from BmNPV infected and un-infected control silkworm larvae were constructed and sequenced using Solexa sequencing. The sequencing data for the two libraries have deposited in the NCBI Small Reads Archive (SRA) database (http://www.ncbi.nlm.nih.gov/sra/) under accession number SRP083131. Parameters of the raw reads including adapter sequences, low quality reads, etc. are shown in S1 Table. A total of 6898880 and 6810268 clean reads (size > 18 nt) were obtained from each small library (Table 1). All identical sequence reads in each small library were grouped and converted into unique reads, which were subsequently mapped onto the silkworm genome using Bowtie. As a result, 620899 and 789310 unique reads were collected from infected and un-infected libraries, respectively (Table 1). More than 40% of the unique reads perfectly matched the silkworm genome. Size distributions of total abundances and unique reads were evaluated and are shown in Fig 1. More than 50% of the small RNAs were 18–23 bp in length with the 20 bp RNAs representing the major length class.

**Table 1:** Classification of sequenced reads from silkworm.

Sample	Raw reads	Clean reads	Unique clean	*Bombyx mori* genome	
				Unique mapped reads	Unique mapped rate (%)
Control	11465648	6898880	620899	297382	47.90
Infection	9579784	6810268	789310	321162	40.69

### Identification of known and novel silkworm miRNAs

**Fig 2:**
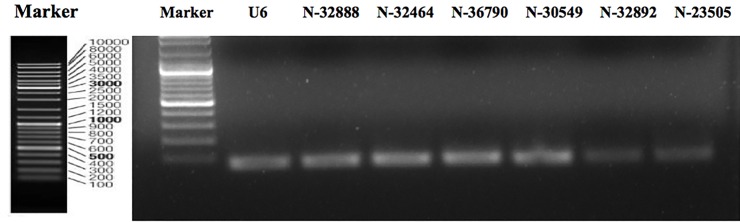
Validation of novel silkworm miRNAs. 1μg total RNA extracted from silkworm larvae was reverse transcribed into cDNA after DNase I treatment. Specific stem-loop primers for 6 novel miRNA were used to perform RT-PCR. The PCR products were separated on agarose gel to detect the expression of novel miRNA. The band sizes were about 70-80bp in length. U6,the *B*. *mori* U6 snRNA gene was as a positive control.

To identify known miRNAs in silkworm, we aligned perfectly mapped unique reads against the silkworm microRNA precursor in the Sanger miRBase (Release 21.0) [26] and obtained 373 known miRNAs. Further, after removing the non-coding RNA (rRNA, tRNA and snoRNA, etc.), multi-mapped reads (>20) and low abundance reads (counts <2), we predicted the secondary structures of the remaining unique reads, which did not find a perfect match in the miRBase (Release 21.0). Those unique reads with a predicted secondary structure were considered as novel miRNAs, among which, those having the same seed sequences with known miRNAs of other species were grouped into conserved miRNAs. As a result, a total of 73 novel miRNAs including 37 conserved miRNAs were identified (Table 2). The details of known and novel miRNAs are shown in S2 and S3 Tables. To validate the newly predicted miRNAs, six novel miRNAs were randomly selected for analysis by stem-loop RT-PCR. Primers used in this experiment are listed in S4 Table. All 6 miRNAs were found to be expressed in the silkworm samples analyzed (Fig 2).

**Table 2:** Summary of miRNAs in two sequenced small RNA libraries.

Sample	Known	Novel	
		Conserved	Unknown
Control	338	32	25
Infection	287	29	18
Total	373	37	36

### Differentially expressed miRNAs are in response to BmNPV infection

**Fig 3:**
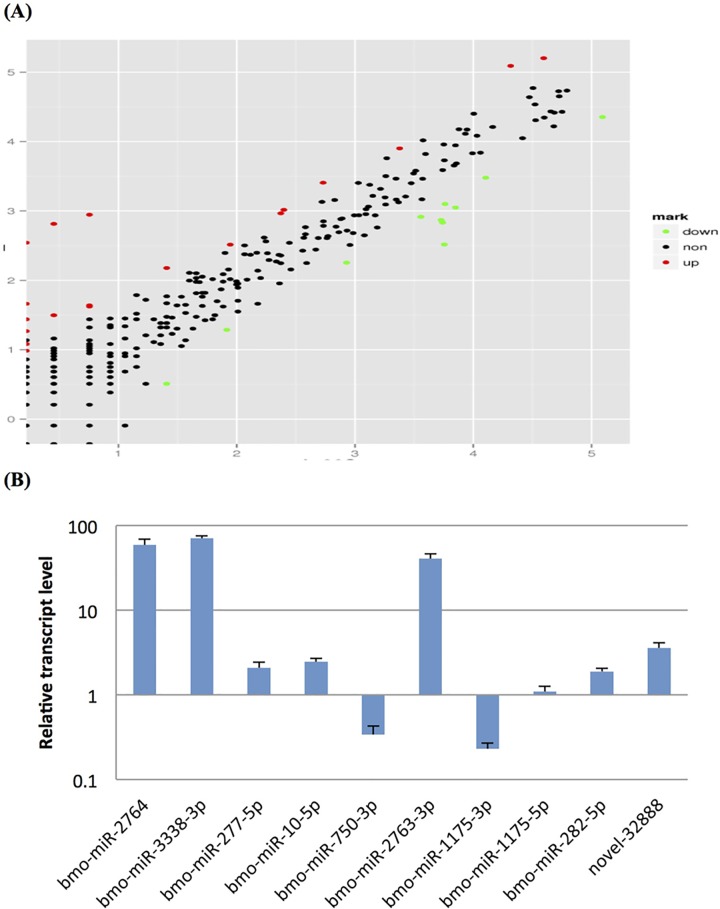
Differentially expressed miRNAs associated with BmNPV infection. (a) The scatter plot of differentially expressed miRNAs. X and Y axes show the normalized expression level of miRNAs in control and infected small libraries, respectively. Red points represent up-regulated miRNAs; Green points represent down-regulated miRNAs. Black points represent non-changed miRNAs. (b) qRT-PCR results of differentially expressed miRNAs. Ct values of each reaction were normalized to U6, the endogenous control. The mean value ± SD was used to analyze relative transcript levels of each miRNA by the 2^-△△Ct^ method. Relative transcript levels of tenmiRNAs in BmNPV infected larvae and non-infection larvae are shown on the left and right, respectively. Error bars represent standard deviation. qRT-PCR reactions were run with three biological replicates in three technical replicates.

The frequency of miRNAs was normalized to TPM (number of transcripts per million clean tags). To investigate whether the silkworm miRNAs were expressed in response to BmNPV infection, we used the following criteria; log_2_FC<-1 or log_2_FC>1 and a P value < = 0.05. This analysis resulted in a total of 38 differentially expressed miRNAs (Fig 3A). Among these, 31 are known miRNAs and 7 are novel miRNAs. A total of 23 up-regulated and 15 down-regulated miRNAs were detected (Table 3). Further analysis revealed that 7 miRNAs including bmo-miR-3338-3p, bmo-miR-3327-3p, bmo-miR-2856-3p, bmo-miR-iab-8, bmo-miR-2828, novel-25022 and novel-7977 were specially expressed in larvae 72h post-BmNPV infection while novel-22398 was expressed in 72h un-infected larvae.

**Table 3:** Differentially expressed miRNAs in BmNPV infected silkworm

miRNA name	Sequences	Length	Control (TPM)	Infection (TPM)	P-Value	Log_2_FC	Mark
bmo-miR-3338-3p	ATGTACTTACTTTGTTTGTTCT	22	0	349.086	0	11.119	up
bmo-miR-3327-3p	ATATGTAACGTTTTTGTTGTCCT	23	0	45.954	0.001	8.198	up
bmo-miR-2764	TTCGTAGATATTGTAGTTACTGG	23	2.843	651.412	0	7.763	up
bmo-miR-2856-3p	ACATTCGAGAACCGTAAGACAA	22	0	27.411	0.007	7.456	up
bmo-miR-2763-3p	TATTATGCTCATTTCTTTGGAT	22	5.686	882.792	0	7.239	up
mo-miR-iab-8	TTACGTATACTGAAGGTATACCG	23	0	12.093	0.033	6.286	up
bmo-miR-2827	CAGACTATCAGTACGTACGCTG	22	0	9.674	0.048	5.968	up
bmo-miR-2733j	AGTCATCATACTCTCAGTTGATA	23	2.843	31.442	0.027	3.397	up
bmo-miR-277-5p	TCGTGCCAGGAGTGCGTTTGC	21	2.843	31.442	0.027	3.397	up
bmo-miR-3325	CAGTGATGAGTCAAATACACCA	22	5.686	43.535	0.026	2.903	up
bmo-miR-282-3p	ACATAGCCTGATAGAGGTTACG	22	5.686	41.923	0.03	2.848	up
bmo-miR-6497-3p	GATGCGGCCGGTGCCGGGTCT	21	20702.36	123236.927	0.004	2.574	up
bmo-miR-7-3p	AAGAAATCACTAATCTGCCTA	21	25.587	150.76	0.01	2.551	up
bmo-miR-277-3p	TAAATGCACTATCTGGTACGACA	23	537.317	2553.243	0.01	2.248	up
bmo-miR-282-5p	ACCTAGCCTCTCCTTGGCTTTGTCTGT	27	250.18	1036.776	0.02	2.05	up
bmo-miR-10-5p	ACCCTGTAGATCCGAATTTGT	21	39397.597	159341.902	0.02	2.016	up
bmo-miR-2733e	TCACTGGGAATGTAATAGCTAT	22	235.965	923.908	0.025	1.968	up
bmo-miR-2733i-3p	TCACTGGGAATGTAATGACTAT	22	88.131	327.318	0.037	1.891	up
bmo-miR-2733c	TCACTGGGAGAGTGATGATTGC	22	2382.392	7972.537	0.042	1.743	up
bmo-miR-6498-5p	CGCGTCTGTTGTCGCAGCCGTGC	23	12744.944	3012.779	0.016	-2.081	down
bmo-miR-2769	ATATATTATCAGATTTTCGGTC	22	82.446	19.349	0.032	-2.082	down
bmo-miR-283-5p	TAAATATCAGCTGGTAATTCT	21	3607.703	823.133	0.014	-2.132	down
bmo-miR-12	TGAGTATTACTTCAGGTACTGGT	23	5768.345	1260.901	0.012	-2.194	down
bmo-miR-2779	ATATCCGGCTCGAAGGACCA	20	847.199	179.783	0.011	-2.235	down
bmo-miR-750-3p	CCAGATCTATCTTTCCAGCT	20	123497.735	22534.167	0.005	-2.454	down
bmo-miR-6497-5p	GCTCTGAGGACCGGGGCGTGTC	22	7084.631	1119.009	0.003	-2.662	down
bmo-miR-1175-5p	AAGTGGAGGTGTGATCTCTTCA	22	5350.431	738.482	0.001	-2.857	down
bmo-miR-283-3p	CAGGCTATCAGCTGGTATACAG	22	25.587	3.225	0.042	-2.928	down
bmo-miR-6498-3p	AACGTCTGCGATGATACAGTT	21	5492.579	679.629	0.001	-3.014	down
bmo-miR-1175-3p	TGAGATTCAACTCCTCCAACTTAA	24	5694.428	328.124	0	-4.117	down
bmo-miR-2819	TCAATGCCTGCTCTATCGGTTC	22	847.219	179.083	0.011	-2.235	down
novel-32892	TCACTGGGAATGTAATAACTAT	22	18393.049	66151.332	0.043	1.844	up
novel-32888	TCACTGGGTATGTAATAGCTAT	22	11318.8	71224.287	0.005	2.649	up
novel-25022	AATTTTCTCGCCGAACCCTTCG	22	0	4870.037	0.027	6.96	up
novel-7977	GCGGCTGTTAACTTTAGGCAGC	22	0	2637.937	0.048	6.086	up
novel-23505	GAGTGTTCGACGGGGTAAC	19	36078.674	5478.791	0.005	-2.71	down
novel-23887	TAATTCGGGGACTTTAGAAAT	21	4951.975	405.836	0.035	-3.487	down
novel-22398	CCTGAGATGATAGGGGATCGCA	22	4244.55	0	0.016	-6.764	down

To confirm the Solexa sequencing results, 10 miRNAs were selected to perform stem loop qRT-PCR assay. One was an identified novel miRNA and the remaining nine were known miRNAs. Among the known miRNAs, six were up-regulated and three were down-regulated. Half of the up-regulated miRNAs had a log_2_FC value >5 and the remaining half had a value between 2 and 5. Except for bmo-miR-1175-5p, which showed no significant difference between BmNPV infected and un-infected larvae, the remaining 9 miRNAs showed an expression pattern similar to the Solexa sequencing data (Fig 3B).

### Gene ontology analysis to predict target genes for differentially expressed miRNAs

**Fig 4:**
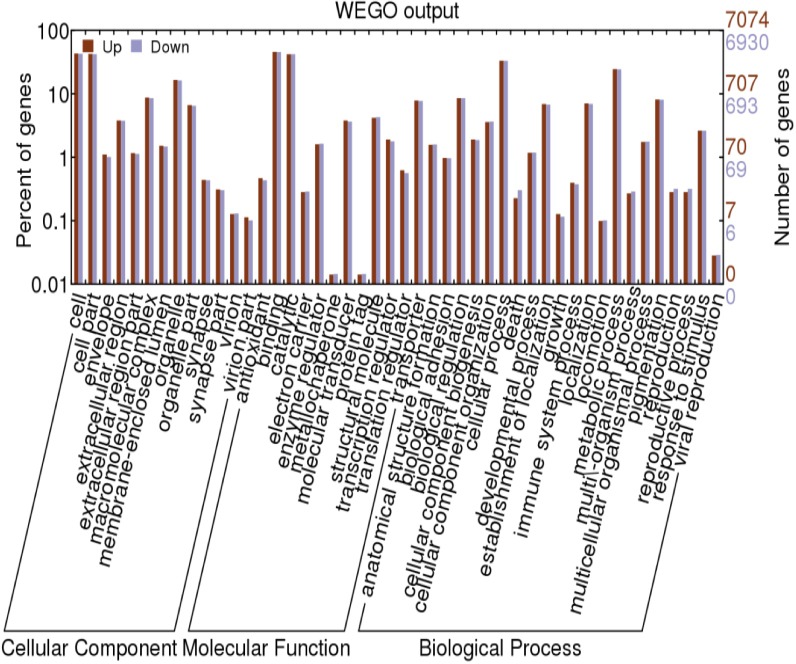
GO categories of the predicted target genes of differentially expressed miRNAs.

GO analysis was performed to those predict target genes for differentially expressed miRNAs. The resulting genes were classified into three categories; cellular component, molecular function and biological process (Fig 4). The top five ranked GO terms were cell, cell part, binding, catalytic and cellular process that together accounted for more than 50%. Nearly 6% of the target genes were involved in virion, virion part, immune system process and response to stimulus.

### Validating the interaction of miR-277-5p with Dnmt2

**Fig 5:**
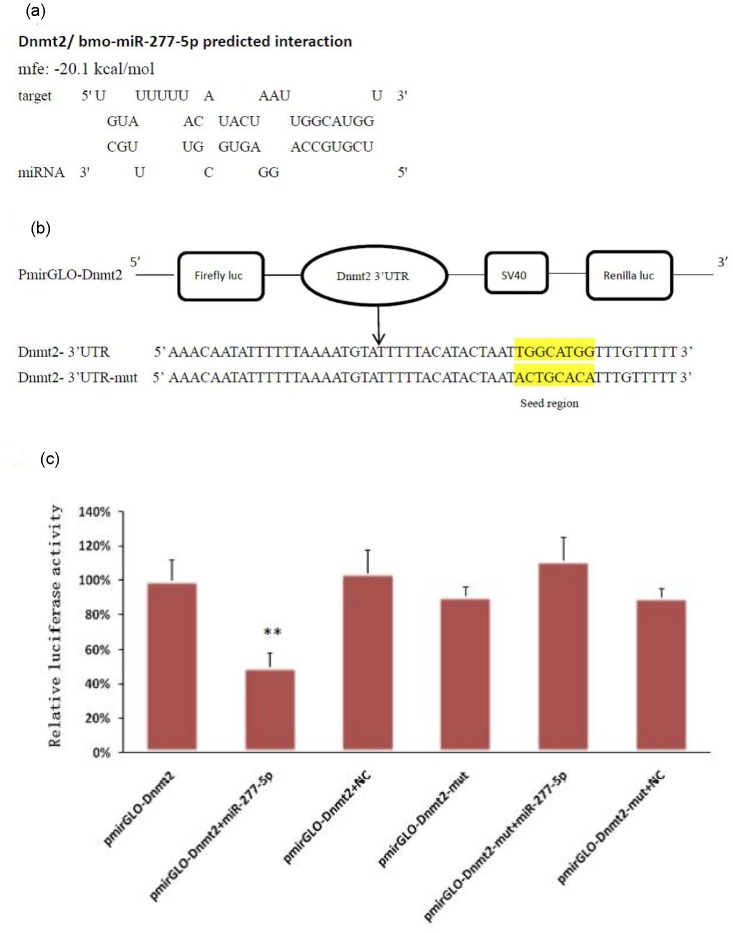
Target validation of miR-277-5p. (a) miR-277-5p was predicted to target Dnmt2. mfe, minimum free energy. (b)Schematic diagram showing the cloning strategy of Dnmt2 target sequence downstream of the firefly luciferase gene in the PmirGLO vector. Nucleotides in the seed region of PmirGLO-Dnmt2 and PmirGLO-Dnmt2-mut constructs are highlighted in yellow. (c) Luciferase reporter assays were carried out in 293 cells, and renilla luciferase was used as an endogenous control. Data are represented as mean ± SD and were generated from three independent experiments performed in triplicates. (**p<0.01).

MiRNA regulates the expression of target gene by imperfect base-pairing to bind seed region in the 3’ UTR of mRNA resulting in mRNA degradation or translation inhibition. Dnmt2 (NM_001043469.1) was predicted to be one of the target genes for miR-277-5p using miRNA seed region binding to the 3’UTR of Dnmt2 mRNA with mfe (minimum free energy) -20.1kcal/mol (Fig 5A). To further validate the positive interaction between miR-277-5p and Dnmt2, dual-luciferase reporter assay was performed. As predicted, we found that the luciferase activity decreased significantly in 293 cells co-transfected with PmirGLO-Dnmt2 and miR-277-5p mimic when compared to the negative control, which indicated that miR-277-5p can directly regulated Dnmt2. To confirm whether miR-277-5P specifically inhibited Dnmt2, the mutated reporter was constructed. Overexpression of miR-277-5p has no effects on luciferase activity in the PmirGLO-Dnmt2-mut transfected cells (Fig 5B and 5C). These results suggested that Dnmt2 was one of the target genes for miR-277-5p.

## Discussion

BmNPV causes severe losses to the sericulture industry. Studies on this virus thus far, have demonstrated its proliferation within the host through a cascade of viral gene expression and identifying host resistant genes against BmNPV infection [27–28]. However, the molecular mechanism of BmNPV-host interaction remains poorly understood. Studies over the years have shown evidences for the contribution of cellular miRNAs as well as viral miRNAs to host immunity and host-pathogen interaction by regulating the expression of host or pathogen related genes in various organisms [29–31]. However, the roles of silkworm miRNAs in BmNPV infection have not been investigated. In order to detect potential silkworm miRNA involved in BmNPV infection, in this study, we constructed two small RNA libraries from BmNPV-infected and un-infected larvae. Small RNA deep sequencing results revealed a total of 373 known and 73 novel silkworm miRNAs, among which, 38 differentially expressed miRNAs in BmNPV-infected silkworm were screened. Then, target gene prediction for these differentially expressed miRNAs was carried out and the results revealed thousands of targets. We analyzed the gene function of all of the predicted targets according to Go analysis attempting to obtain a glancing description on potential genes related to BmNPV infection. The results showed that the majority of predicted target genes were involved in cell, binding, catalytic, and cellular process, which is consistent with the results of miRNAs response to BmCPV infection [19]. Meanwhile, we also found a small amount of target genes were involved in immune system process, response to stimulus and virion part indicating these genes and the correspondent miRNAs may have potential roles in silkworm-BmNPV interaction and need to be studied in the future.

Subsequently, we confirmed differential expression of 9 miRNAs in BmNPV-infected silkworm larvae using stem-loop qRT-PCR. Out of these, we found that the expression of miR-282 was induced by BmNPV infection. MiR-282 is reported to regulate viability, longevity and egg production in *Drosophila*. The nervous system-specific adenylate cyclase is a target of miR-282, and one of the main functions of miR-282 is the regulation of adenylate cyclase activity in the nervous system during metamorphosis [32]. Ame-miR-282, encoded by *Apis mellifera L*., is down regulated in dancing honey bees compared to foraging honey bees. The target genes of ame-miR-282 were also found to be associated with kinase, neural function, synaptotagmin and energy indicating that ame-miR-282 could play a role in regulating the dancing behaviors in honey bees [33]. In this study, up-regulation of miR-282 upon BmNPV infection suggesting miR-282 may be involved in BmNPV infection.

In addition, miR-10-5p was also found to up-regulate in BmNPV infected silkworm larvae indicating that miR-10-5p may also play a role in BmNPV infection. *Spodoptera exigua* miR-10-1a has been shown to be differentially expressed during the larval stages. Oral feeding experiments using synthetic miR-10-1a mimic can suppress the growth of *S*. *exigua* and increased mortality [34].

Among the 9 differential expressed miRNA, we found several studies have reported the potential functions of miR-277. For example, miR-277 expression was induced in response to parasitization by the *Glyptapanteles flavicoxis* parasitoid [35]. *Drosophila* miR-277 is down regulated during the adult life, and controls branched-chain amino acid catabolism. Constitutive miR-277 expression shortens lifespan [36] and plays a role as a metabolic switch controlling amino acid catabolism by targeting the metabolic pathway enzymes [37]. MiR-277 has been also reported to be associated with freeze tolerance in insects [38] and to modulate rCGG repeat-mediated neurodegeneration [39].

In this study, we found that miR-277-5p was significantly up-regulated in BmNPV infected silkworm larvae. To explore whether miR-277-5p play roles in silkworm-BmNPV interaction, we investigated the target gene of miR-277-5p. Based on bioinformatics prediction and dual-luciferase reporter assay, we confirmed the positive interaction between miR-277-5p and Dnmt2. Dnmt2 is both a cytoplasmic and a nuclear protein [40] that specially methylating cellular tRNAs. Cytosine-5 methyltransferases of the Dnmt2 family are highly conserved through evolution and their biological function has been studied in several organisms. For instance, Dnmt2 is required for an efficient innate immune response in *Drosophila*. Dnmt2 mutant flies accumulate increasing levels of *Drosophila* C virus and show activated innate immune responses [41]. Overexpression of Dnmt2 can prolong the life span of *Drosophila* and increased the resistance to stress [42]. *Drosophila* Dnmt2 loss-of-fuction mutants showed decreased viability under stress conditions [43]. Dnmt2 is also essential for the efficient functioning of Dicer-2 (Dcr-2) in *Drosophila* [44]. Infection with *Wolbachia* can disrupt the genome-wide patterns of cytosine methylation in *Aedes aegypti* [45]. Mosquitoes infected with the dengue virus induces expression of Dnmt2 while when mosquitoes introduced endosymbiont, *Wolbachia*, the expression of Dnmt2 is significantly decreased. Interestingly, overexpression of Dnmt2 in mosquito cells led to inhibition of *Wolbachia* replication, but significantly promoted replication of dengue virus, suggesting a causal link between this *Wolbachia* manipulation and the blocking of dengue replication in *Wolbachia* infected mosquitoes [46]. Together, these studies implicated that Dnmt2 is functional for cellular stress responses at least in adult flies and mosquito.

In the present study, we speculated that BmNPV infection induced the expression of miR-277-5p, which led to regulate the expression of Dnmt2 gene. Cleavage of tRNA is a conserved response to several stress stimuli in eukaryotes [47]. Dnmt2-mediated methylation protected tRNA from stress-induced ribonuclease cleavage [43]. Down-regulation of Dnmt2 caused reduced methylation of cellular tRNAs, as a result, some tRNA frgaments are produced to repress translation by displacing translation initiation and elongation from mRNAs or by interfering with efficient transpeptidation [41]. Further experiments are necessary to demonstrate the precise function of Dnmt2 in BmNPV-host interaction.

In summary, the present study provides the firstly genome-wide characterization of miRNAs in BmNPV infected silkworm. The differentially expressed miRNAs between BmNPV-infected and un-infected silkworm are expected to offer new insights to explore the molecular mechanisms underlying the BmNPV-host interactions. Further studies are necessary to validate the function of the related miRNAs and predicted target genes in silkworm.

## Supporting Information

S1 Table Expression profile of sequenced reads in two libraries. (DOC)

S2 Table known miRNAs in two small RNA libraries. (DOCX)

S3 Table novel miRNAs identified in two small libraries. (DOCX)

S4 Table Primer sequences used in this study. (DOCX)
