# Supplementary material for: Genome-Wide Analysis of Differentially Expressed microRNA in Bombyx mori Infected with Nucleopolyhedrosis Virus
Source: PLoS One. 2016 Nov 2;11(11):e0165865. doi: 10.1371/journal.pone.0165865 (PMC5091789; doi:10.1371/journal.pone.0165865)
Supplement: S1 Table — (DOC) [file pone.0165865.s001.doc]

S1 Table. Expression profile of sequenced reads in two libraries

| Sample_ID | Raw_reads | Adapter (%) | Low_quality(%) | Too-short(%) | Too-long(%) | Clean_reads(%) | Uniq_clean_reads(%) |
| --- | --- | --- | --- | --- | --- | --- | --- |
| Control | 11465648 | 0.03 | 0.28 | 36.01 | 3.49 | 60.17 | 9 |
| Infection | 9579784 | 0.27 | 0.22 | 25.3 | 3.09 | 71.09 | 11.59 |
