# Supplementary material for: Genome-Wide Analysis of Differentially Expressed microRNA in Bombyx mori Infected with Nucleopolyhedrosis Virus
Source: PLoS One. 2016 Nov 2;11(11):e0165865. doi: 10.1371/journal.pone.0165865 (PMC5091789; doi:10.1371/journal.pone.0165865)
Supplement: S2 Table — (DOCX) [file pone.0165865.s002.docx]

S2 Table. Known miRNAs in two small RNA libraries

| miRNA name | Sequence | Length | Control | Infection |
| --- | --- | --- | --- | --- |
| bmo-miR-3213-3p | TGATATGACCGAGTGTTTGTTAGAAA | 26 | 0 | 1.612 |
| bmo-miR-2808a-5p | CGTAGAATCTACCATCGGATCG | 22 | 42.644 | 20.155 |
| bmo-miR-2723 | CCAGCAGTGGACGTCTGTG | 19 | 2.843 | 0.806 |
| bmo-miR-927-3p | CAAAGCGTTTGGATTCTAA | 19 | 62.545 | 104 |
| bmo-miR-307-3p | TCACAACCTCCTTGAGTGAG | 20 | 7567.932 | 15041.321 |
| bmo-miR-2830 | AGCGACTATCTCAGGGCGCGCT | 22 | 31.272 | 43.535 |
| bmo-miR-2805 | TTCCTGACGAACCACGGAAA | 20 | 8.529 | 2.419 |
| bmo-miR-970-3p | TCATAAGACACACGCGGCTCT | 21 | 32014.457 | 59047.081 |
| bmo-miR-6498-3p | AACGTCTGCGATGATACAGTT | 21 | 5492.579 | 679.629 |
| bmo-miR-2807b | CAACTGTTTCAACTCCATACAA | 22 | 5.686 | 0.806 |
| bmo-miR-1175-5p | AAGTGGAGGTGTGATCTCTTCA | 22 | 5350.431 | 738.482 |
| bmo-miR-2756-5p | ACCCTGTAGCTGCCAAGGGGCG | 22 | 1239.526 | 1059.35 |
| bmo-miR-263b-5p | CTTGGCACTGGGAGAATTCAC | 21 | 25.587 | 58.853 |
| bmo-miR-2824 | AACTTCAAACCGCCACAGGTAACC | 24 | 8.529 | 8.868 |
| bmo-miR-276-5p | AGCGAGGTATAGAGTTCCTACG | 22 | 1816.645 | 1238.327 |
| bmo-miR-13b-3p | TATCACAGCCATTTTTGACGAGT | 23 | 730.638 | 493.396 |
| bmo-miR-7-3p | AAGAAATCACTAATCTGCCTA | 21 | 25.587 | 150.76 |
| bmo-miR-2769 | ATATATTATCAGATTTTCGGTC | 22 | 82.446 | 19.349 |
| bmo-miR-11-5p | TAGCACTCGACATGTGACCTGT | 22 | 591.334 | 433.737 |
| bmo-miR-2758-5p | ACTTGGTAGAACACGTAGTAAG | 22 | 5694.428 | 5364.472 |
| bmo-miR-2834 | TAATATCGGGACTAGACGAC | 20 | 2.843 | 7.256 |
| bmo-miR-279c-5p | GGGCGAGTTTGCTTCTGGTTCA | 22 | 34.115 | 58.047 |
| bmo-miR-279a | TGACTAGATCCACACTCAT | 19 | 5631.883 | 9089.934 |
| bmo-miR-2771-5p | TAACATTACGAGGATGGGTTTAGTTA | 26 | 2.843 | 0 |
| bmo-miR-3246 | TTGCTATCAGAACATTGTGTCAT | 23 | 0 | 0.806 |
| bmo-miR-3204a-5p | CGCGTCATAAACTTCCCATAT | 21 | 11.372 | 21.767 |
| bmo-miR-3267c | GACTGAGCTCGTCCATTCATCT | 22 | 0 | 0 |
| bmo-miR-2816 | CTCGAGTGAGGTAGAGTCGT | 20 | 0 | 0.806 |
| bmo-miR-3204b | TGCGTCATAAACTTCCCATAT | 21 | 0 | 0.806 |
| bmo-miR-2857 | TGATGGGACACATACGGAACAT | 22 | 0 | 0.806 |
| bmo-miR-3372-3p | CGCCAAGCTATTTTAAGCAATGA | 23 | 0 | 4.031 |
| bmo-miR-2739 | GAGATGTGGATATTATGGTGTGGAGG | 26 | 2.843 | 3.225 |
| bmo-miR-3327-3p | ATATGTAACGTTTTTGTTGTCCT | 23 | 0 | 45.954 |
| bmo-miR-929-3p | CTCCCTAATCGAGTCAGGTTGA | 22 | 8.529 | 4.031 |
| bmo-miR-3402 | TGCAATAACTTTAAACCGCTGAGCT | 25 | 0 | 1.612 |
| bmo-miR-3238 | AAATGAATCATGCATTGACTACATG | 25 | 8.529 | 3.225 |
| bmo-miR-2774a | CTCGGTGGAAGATCGTCCCT | 20 | 1643.225 | 2080.003 |
| bmo-miR-281-3p | ACTGTCATGGAGTTGCTCTCTT | 22 | 3681.62 | 1471.32 |
| bmo-miR-2797a | ATAAGTAGGTTTTATTCGACTT | 22 | 2.843 | 0 |
| bmo-miR-133 | TTGGTCCCCTTCAACCAGCTGT | 22 | 369.583 | 410.357 |
| bmo-miR-iab-8 | TTACGTATACTGAAGGTATACCG | 23 | 0 | 12.093 |
| bmo-miR-282-3p | ACATAGCCTGATAGAGGTTACG | 22 | 5.686 | 41.923 |
| bmo-miR-3324 | TCCATGATCCCTGAGTGCCTAGGT | 24 | 34.115 | 11.287 |
| bmo-miR-190-5p | AGATATGTTTGATATTCTTGGTT | 23 | 466.244 | 277.334 |
| bmo-miR-2b-5p | TCGACAAGGTGGTTGTGACATG | 22 | 380.955 | 461.954 |
| bmo-miR-2798 | TGTGCGACAGAGATAGCGCTCG | 22 | 0 | 0 |
| bmo-miR-3303 | TTGGATTACGCCCTCTGACACCTC | 24 | 0 | 0.806 |
| bmo-miR-281-5p | AAGAGAGCTATCCGTCGACAGT | 22 | 47917.917 | 16570.687 |
| bmo-miR-3347b | TACATCATTGATCTTGCTATCTCC | 24 | 0 | 0.806 |
| bmo-miR-316-5p | TGTCTTTTTCCGCTTTGCTGCTG | 23 | 1199.725 | 445.024 |
| bmo-miR-2843-2-3p | TTCGTGATCAAGCCTGACCCCTTAAT | 26 | 5.686 | 8.868 |
| bmo-miR-2772b | TGTTTGAGTTCTGGATCTGGA | 21 | 8.529 | 4.837 |
| bmo-miR-2755-3p | CACCCTGTCAGACCATACTTGTT | 23 | 2231.716 | 1456.808 |
| bmo-miR-278-5p | CCGGACGAACTTCCCAGCTCGG | 22 | 39829.725 | 22168.958 |
| bmo-miR-2733b | TCACTGGGTGCGTGATGATTGT | 22 | 2.843 | 8.868 |
| bmo-miR-305-5p | ATTGTACTTCATCAGGTGCTCTG | 23 | 2985.097 | 2512.127 |
| bmo-miR-31-3p | GGCTGTGTCACTTCGAGCCAGC | 22 | 51.173 | 42.729 |
| bmo-miR-2765 | TGGTAACTCCACCACCGTTGGC | 22 | 17.058 | 3.225 |
| bmo-miR-306a-3p | CAGAGCCGCCTCGTGCCTCAG | 21 | 73.917 | 74.171 |
| bmo-miR-2733d | TCACTGGGTGTATGAAGATTGT | 22 | 8.529 | 22.574 |
| bmo-miR-3275 | TGATTCTTGAGTAGGACTTCCTTC | 24 | 2.843 | 4.837 |
| bmo-miR-2778b-5p | GTTTTTTTGCATATCCTGCA | 20 | 230.279 | 90.295 |
| bmo-miR-10-3p | CAAATTCGGTTCTAGAGAGGTTT | 23 | 986.504 | 865.055 |
| bmo-miR-2831 | TACAAACAAAGCATTTGTTTGA | 22 | 2.843 | 3.225 |
| bmo-miR-2999 | CTGCGACGGACTAGACGCGCA | 21 | 51.173 | 112.868 |
| bmo-miR-87 | TGAGCAAACTTTCAGGTGTGT | 21 | 181.949 | 245.892 |
| bmo-miR-2744 | TTTGAGAGTCCTAGCTAG | 18 | 2.843 | 2.419 |
| bmo-miR-3344 | TTGCAAGAAGGACTCAGCCAGCGAG | 25 | 2.843 | 3.225 |
| bmo-miR-993a-3p | GAAGCTCGTCTCTACAGGTATCT | 23 | 5.686 | 11.287 |
| bmo-miR-2758-3p | CCCTGCGTGTTCTACCAAGTTA | 22 | 540.16 | 610.295 |
| bmo-miR-3383-3p | CCACGAAGTTGACGCGCTGTCTCT | 24 | 0 | 0.806 |
| bmo-miR-184-5p | CCTTGTCATTCTTCAGGCCCTG | 22 | 116.561 | 237.83 |
| bmo-miR-2813 | TTCTAGATTAAGCCGTTGGAA | 21 | 8.529 | 8.868 |
| bmo-miR-2808a-3p | CGGTGGTAGATTCTGCGAAGTACG | 24 | 45.487 | 65.302 |
| bmo-miR-2780b | AAGGGTGTTAAATGAAGACTTAA | 23 | 28.429 | 29.023 |
| bmo-miR-2748 | TCCGGCGAGAAAACTCAGTGGG | 22 | 0 | 1.612 |
| bmo-miR-9c-3p | TAAAGTTATGGTACCGAAGTTA | 22 | 1233.84 | 899.722 |
| bmo-miR-2835 | TCACACATTATGAGCTTTAGGA | 22 | 2.843 | 9.674 |
| bmo-miR-13a-5p | CCTGTCAAAGCGGCGGTGAAA | 21 | 8.529 | 8.868 |
| bmo-miR-3296-5p | TTTTGAGTATGCCCGCTGTACAATTCC | 27 | 0 | 0 |
| bmo-miR-3204a-3p | TATGGGAGTTTATGACTTAGG | 21 | 0 | 3.225 |
| bmo-miR-13a-3p | TATCACAGCCACTTTGATGTG | 21 | 227.436 | 232.993 |
| bmo-miR-989a | GTGTGATGTGACGTAGTGGAA | 21 | 2.843 | 0.806 |
| bmo-miR-2860 | CGCTGTATGATTGTCCGAGGTA | 22 | 8.529 | 12.093 |
| bmo-miR-3268-5p | TGAAGTATGATTCATTAGTAGACTA | 25 | 2.843 | 1.612 |
| bmo-miR-2733a-3p | AGTCATCACACTCTCAGTTGATA | 23 | 22.744 | 20.961 |
| bmo-miR-929-5p | ATTGACTCTAGTAGGGAGTCC | 21 | 8.529 | 3.225 |
| bmo-miR-3406-3p | TCGCGTGTTAGACAAGCTGCGCCATGCT | 28 | 5.686 | 4.031 |
| bmo-miR-2780c | AAGGGCGTTAAATGAAGACTTAG | 23 | 62.545 | 27.411 |
| bmo-miR-3257-3p | TACGTTATGAGTCGTCGCGTTAA | 23 | 2.843 | 0 |
| bmo-miR-2757-3p | CTGAAAGTGTCCATTTCCGTT | 21 | 85.288 | 143.504 |
| bmo-miR-2778a-5p | GTTTTTTTGCATATCCTGCA | 20 | 230.279 | 90.295 |
| bmo-miR-3345 | TATCGTTACGCGCGTCGCTTAGG | 23 | 2.843 | 0 |
| bmo-miR-3330 | TACCGTAGTGAAAGCGCTCCACGA | 24 | 0 | 0 |
| bmo-miR-3244 | TAGCTGACGATGGAATCTTTCTA | 23 | 0 | 0.806 |
| bmo-bantam-3p | TGAGATCATTGTGAAAGCTAATT | 23 | 56344.42 | 26813.49 |
| bmo-miR-3385-3p | TGACTGTTTTGGTGTATGGTAGAG | 24 | 0 | 0 |
| bmo-miR-279b-5p | ATGGGTATAGGTCTAGTAT | 19 | 2675.216 | 1613.211 |
| bmo-miR-745-3p | CAGCTGCCTAGCGAAGGGCAACG | 23 | 29779.898 | 43631.682 |
| bmo-miR-3308-5p | TACTTTATCTTGGTAATTGGTTGTC | 25 | 5.686 | 12.093 |
| bmo-miR-3229 | TTGTAGTATCTTCCTTGATACCGGA | 25 | 0 | 4.837 |
| bmo-miR-3254 | GCGGACGAACTTAAAGAAGAG | 21 | 0 | 2.419 |
| bmo-miR-2839-3p | CGCTGGCGCACTGTTTGACGGT | 22 | 0 | 0 |
| bmo-miR-2775b | AGTAGGACGTTTCCTCCCGCGTG | 23 | 5.686 | 3.225 |
| bmo-miR-3406-5p | TAGTAAGTAGCACGTTTGATGAGCA | 25 | 2.843 | 10.481 |
| bmo-miR-307-5p | ACTCACTCAACCTGGGTGTGATG | 23 | 14.215 | 61.271 |
| bmo-miR-6497-5p | GCTCTGAGGACCGGGGCGTGTC | 22 | 7084.631 | 1119.009 |
| bmo-miR-3317 | TACAGGAATGTTGTGATAAGCTCTG | 25 | 2.843 | 3.225 |
| bmo-miR-3309 | TGTAATAGTATAATTGTGCTCTTG | 24 | 0 | 3.225 |
| bmo-miR-3296-3p | TAGCTACTCGGGATATGCTGTCTTT | 25 | 8.529 | 5.643 |
| bmo-miR-3294 | ACCACAGACAGTATCTAACCTGA | 23 | 2.843 | 0 |
| bmo-miR-2774b | CTCGGTAGAAGATCTTCCCTTT | 22 | 778.968 | 778.792 |
| bmo-miR-2843-1-3p | TTTGCGACGCTGTCTTCGTGATCAA | 25 | 11.372 | 4.837 |
| bmo-miR-2840 | TAGGAACTGGAAGAAGAGGAGG | 22 | 2.843 | 2.419 |
| bmo-miR-2755-5p | CAAGGTGGCCTAGCAGAGTGTT | 22 | 14.215 | 10.481 |
| bmo-miR-6497-3p | GATGCGGCCGGTGCCGGGTCT | 21 | 20702.36 | 123236.927 |
| bmo-miR-375-3p | TTTGTTCGCCCCGGCTCGTGTCG | 23 | 235.965 | 228.155 |
| bmo-miR-3350 | TCATGTATTCTAATTTTTCTTTATA | 25 | 36.958 | 13.705 |
| bmo-miR-2776 | AGAGTACGCAAAAAAACAATT | 21 | 5509.637 | 3883.478 |
| bmo-miR-3271 | TGACATCATGTGCGCGTGCTGCT | 23 | 8.529 | 21.767 |
| bmo-miR-2833 | AGAGATTGGAAATGTACTTTTTG | 23 | 11.372 | 14.512 |
| bmo-miR-71-3p | TTCTCACTACCTTGTCTTTCAT | 22 | 3130.088 | 3467.477 |
| bmo-miR-3334 | TGAACCAGAATGATGGAAGGACAG | 24 | 176.263 | 364.404 |
| bmo-miR-2804-3p | TTTGCATTGTAATACACTGTTA | 22 | 102.346 | 35.473 |
| bmo-miR-2788 | TGGGGTTTCAAAGCGGTATGTG | 22 | 0 | 1.612 |
| bmo-miR-2751 | GTCTGGGCCGTGGAGCGTTT | 20 | 2.843 | 0.806 |
| bmo-miR-2770-5p | TAGATAGAATTCGTCGGTGGATAA | 24 | 45.487 | 108.031 |
| bmo-miR-33-3p | CAATATGACTACAAGGCAAATC | 22 | 389.484 | 177.365 |
| bmo-miR-6498-5p | CGCGTCTGTTGTCGCAGCCGTGC | 23 | 12744.944 | 3012.779 |
| bmo-miR-3375-3p | TGCTATCTTTGAAAAGTTTGGAATA | 25 | 0 | 0 |
| bmo-miR-3342 | TGACCATGGTGACGAGTCGAGCCC | 24 | 2.843 | 0 |
| bmo-miR-2b-3p | TATCACAGCCAGCTTTGTTGAGT | 23 | 1793.901 | 1567.258 |
| bmo-miR-3384-5p | TGAAATGTGTCCTCTATCCGGCTC | 24 | 2.843 | 0 |
| bmo-miR-3405 | TCCATTGTGTTCTTGAAACTCTCTA | 25 | 278.609 | 347.473 |
| bmo-miR-3340 | TATACGGATGCGATGACGATCGGCT | 25 | 14.215 | 33.054 |
| bmo-miR-3325 | CAGTGATGAGTCAAATACACCA | 22 | 5.686 | 43.535 |
| bmo-miR-2854 | TCTTTCAAAACTGCAGGACGG | 21 | 5.686 | 13.705 |
| bmo-miR-2733e | TCACTGGGAATGTAATAGCTAT | 22 | 235.965 | 923.908 |
| bmo-miR-3326 | TAGGTTTTGTGGTTCTTGTGAAATA | 25 | 2.843 | 4.031 |
| bmo-miR-2787-5p | TATCTTGAATTTGATTAGCACTAAA | 25 | 22.744 | 24.186 |
| bmo-miR-3321 | CAGAAGTCAAGCATCGCCTTCCA | 23 | 5.686 | 20.961 |
| bmo-miR-3257-5p | TGGCGATTACGCGATTTGCCGTGACCAGA | 29 | 2.843 | 2.419 |
| bmo-miR-283-5p | TAAATATCAGCTGGTAATTCT | 21 | 3607.703 | 823.133 |
| bmo-miR-2838 | AATTCAGCAAACTCACGGGATAA | 23 | 5.686 | 1.612 |
| bmo-miR-3327-5p | AACAACAGGAATGTTATGTAC | 21 | 0 | 0.806 |
| bmo-miR-3318 | GGATTTTACATTTTTGCATGTTGC | 24 | 0 | 0.806 |
| bmo-miR-2797b | ATAAGTAGGTTCTTATTCGGCT | 22 | 2.843 | 4.837 |
| bmo-miR-2807c-5p | TACGACGATGCGACAAATATGACA | 24 | 19.901 | 12.899 |
| bmo-miR-9a-3p | ATAAAGCTAGGTTACCGGAGTTA | 23 | 79.603 | 248.31 |
| bmo-miR-745-5p | CGGCTCATCGTGTGGCAGTTTGCT | 24 | 10746.35 | 12123.674 |
| bmo-miR-9a-5p | TCTTTGGTTATCTAGCTGTATGA | 23 | 1856.446 | 5754.674 |
| bmo-miR-2801 | GTGGAATCTTGTGACTTTGTAAAG | 24 | 540.16 | 707.846 |
| bmo-miR-3377-5p | TGAAGTTATGAAGTCAGGATA | 21 | 0 | 0 |
| bmo-miR-3364 | TTCCGTGACTATCACTTCCATAAT | 24 | 0 | 1.612 |
| bmo-miR-2738 | ATCTTTTAGAACGGCCATCTGATG | 24 | 76.76 | 122.543 |
| bmo-miR-3389-5p | TCGTAGCCGATGTTCCACAGCAG | 23 | 0 | 1.612 |
| bmo-miR-3248 | TGTAAATCAAATGTGTACACCTG | 23 | 0 | 0.806 |
| bmo-miR-2778c-3p | CAGAGTACGCAAAAAATCAATT | 22 | 0 | 0 |
| bmo-miR-275-5p | CGCGCTACTCCGGCGCCAGGACT | 23 | 6874.252 | 4517.959 |
| bmo-miR-3219 | GTGAGACTAATATATCCATGGTGT | 24 | 99.503 | 96.744 |
| bmo-miR-263a-5p | AATGGCACTGGAAGAATTCAC | 21 | 10103.843 | 25179.318 |
| bmo-miR-2778c-5p | GTTTTTTTGCATATCCTGCA | 20 | 230.279 | 90.295 |
| bmo-miR-2733j | AGTCATCATACTCTCAGTTGATA | 23 | 2.843 | 31.442 |
| bmo-miR-279b-3p | TGACTAGATCTACACTCATTGA | 22 | 7198.349 | 4838.828 |
| bmo-miR-317-3p | AGTGAACACAGCTGGTGGTATC | 22 | 3946.014 | 6621.342 |
| bmo-miR-2792-5p | TGGTATCGTTCTTTAATCAACA | 22 | 5.686 | 0 |
| bmo-miR-2809 | GAAACAGCGAACGGATCACCTGAT | 24 | 76.76 | 95.938 |
| bmo-miR-3391-3p | TGACAAAAAGTGATGTGAGTCCGTC | 25 | 2.843 | 5.643 |
| bmo-miR-2856-3p | ACATTCGAGAACCGTAAGACAA | 22 | 0 | 27.411 |
| bmo-miR-2756-3p | CCCCTCGGCTGCTACATTGTAT | 22 | 25.587 | 20.961 |
| bmo-miR-2808b | CGTAGAATCTACCATCGGATC | 21 | 22.744 | 12.093 |
| bmo-miR-3414-5p | TCCTTAAGTTTTTTGAACGCAGTG | 24 | 5.686 | 9.674 |
| bmo-miR-3268-3p | GTGTTTGTCGATGAATTATTGGTACTCA | 28 | 0 | 0 |
| bmo-miR-2848 | TTCGCGGCAGAAATAGGCAAA | 21 | 5.686 | 9.674 |
| bmo-miR-3263 | TAGCTGTTGAGTCCCGTTGAGAA | 23 | 14.215 | 27.411 |
| bmo-miR-305-3p | GGCGCTTGTTGGAGTACACTT | 21 | 216.064 | 187.039 |
| bmo-miR-1b-3p | TGGGAAGTAAGGAAGCACGGAA | 22 | 2.843 | 0.806 |
| bmo-miR-iab-4-5p | ACGTATACTGAATGTATCCTGA | 22 | 150.676 | 137.861 |
| bmo-miR-3375-5p | TCCATGTGTAATTCTTCTTGGT | 22 | 0 | 0.806 |
| bmo-miR-3355 | TAATACTATAAAGGTTCAAGTCA | 23 | 0 | 4.031 |
| bmo-miR-2754 | TCTGGTATTGACTGTAATAG | 20 | 5.686 | 4.837 |
| bmo-miR-11-3p | CATCACAGTCAGAGTTCTAGCT | 22 | 7070.416 | 8810.988 |
| bmo-miR-2764 | TTCGTAGATATTGTAGTTACTGG | 23 | 2.843 | 651.412 |
| bmo-miR-2760-3p | TTTTTGGTTGTTAAGTTCCATT | 22 | 361.055 | 267.659 |
| bmo-miR-998 | TAGCACCATGGGATTCAGCT | 20 | 170.577 | 413.582 |
| bmo-miR-2760-5p | CGAGGCTTAATTGAACCAAAAAGC | 24 | 150.676 | 45.954 |
| bmo-miR-993a-5p | TCTACCCTGTAGATCCGGGCTTTT | 24 | 2.843 | 8.062 |
| bmo-miR-3203-3p | ATGTCAGCTCAGTCAGTACACG | 22 | 11.372 | 8.868 |
| bmo-miR-2819 | TCAATGCCTGCTCTATCGGTTC | 22 | 847.219 | 179.803 |
| bmo-miR-2807c-3p | TCATATTTCGCATTGTTGACG | 21 | 22.744 | 16.124 |
| bmo-miR-2780a-5p | AAGGGTGTTAAATGAAGACTTAT | 23 | 28.429 | 29.023 |
| bmo-miR-2797c | ATAAGTAGGTTTTATTCAGTTT | 22 | 2.843 | 0 |
| bmo-miR-2a-2-5p | CTCACAAAGTGGTTGTCGTATG | 22 | 36.958 | 33.861 |
| bmo-miR-2791 | TTTTCATAACAGCTCTGTCAGC | 22 | 11.372 | 28.217 |
| bmo-miR-2780d | AAAGGTGTTAAATGAAGACTTAG | 23 | 65.388 | 31.442 |
| bmo-miR-1a-3p | TGGAATGTAAAGAAGTATGGAG | 22 | 579.962 | 406.326 |
| bmo-miR-274-5p | GTTTGTGACCGTCACTAACGGGCAGT | 26 | 45285.346 | 27154.514 |
| bmo-miR-2757-5p | ACGGGAATGGTCACTTACAACT | 22 | 45.487 | 126.574 |
| bmo-miR-2796-3p | GTAGGCCGGCGGAAACTACTTGC | 23 | 0 | 0 |
| bmo-miR-9d | ATAAAGCTAATTCACTGAGTGT | 22 | 147.833 | 249.923 |
| bmo-miR-2761-5p | CATCGAGACGGTTCCATACA | 20 | 2.843 | 14.512 |
| bmo-miR-252-3p | CCTGCTGCCTAAGTGCTTATCA | 22 | 0 | 5.643 |
| bmo-miR-3230 | CAGTAGTGAAATGGGAGTCCATGT | 24 | 0 | 0.806 |
| bmo-miR-10-5p | ACCCTGTAGATCCGAATTTGT | 21 | 39397.597 | 159341.902 |
| bmo-miR-2767 | CAAGTAAATCTCGTGCGGTTTG | 22 | 380.955 | 583.691 |
| bmo-miR-3295 | GATCGAGAAGTTAAATAACTCGC | 23 | 2.843 | 1.612 |
| bmo-miR-2772a | TGTTTGAGTTCTGGACCTGGA | 21 | 2.843 | 8.062 |
| bmo-miR-3399 | TATCTACATTGAATACCTGGCTATG | 25 | 25.587 | 24.186 |
| bmo-miR-2733c | TCACTGGGAGAGTGATGATTGC | 22 | 2382.392 | 7972.537 |
| bmo-miR-2778d-5p | GTTTTTTTGCATATCCTGCA | 20 | 230.279 | 90.295 |
| bmo-miR-3336 | TCTGTGGTTGCGTGATCGCCGAG | 23 | 2.843 | 2.419 |
| bmo-miR-3396 | TCCAGTTTCTATTTCACGGACGTCA | 25 | 0 | 0.806 |
| bmo-miR-3351 | TTACGTTGTGGATGTCTATGGGC | 23 | 68.231 | 49.985 |
| bmo-miR-2774c | CTCGGTGGAAGACCTTCCCTT | 21 | 756.225 | 763.474 |
| bmo-miR-2789 | GTAGAAAGTTTGGAGGTGGAT | 21 | 0 | 0.806 |
| bmo-miR-2a-1-5p | GCATCAAAGTCGGTTTGTCATA | 22 | 1077.478 | 866.668 |
| bmo-miR-2733a-5p | TCACTGGGTGCATGATGATTGT | 22 | 25.587 | 46.76 |
| bmo-miR-276-3p | TAGGAACTTCATACCGTGCTCT | 22 | 53245.605 | 44878.071 |
| bmo-miR-3382-5p | TCAAGTTTTGTGTTCTGGGTCGT | 23 | 17.058 | 16.124 |
| bmo-miR-263b-3p | GTGAATTTCCCGATGCCTTAG | 21 | 5.686 | 27.411 |
| bmo-miR-308-3p | AATCACAGGATAATACTGCGAG | 22 | 2220.344 | 2919.259 |
| bmo-miR-3228 | GAGATCAAATGTTGCTGTCCTTGGA | 25 | 2.843 | 0 |
| bmo-miR-2779 | ATATCCGGCTCGAAGGACCA | 20 | 847.199 | 179.783 |
| bmo-miR-2775a | CGCGGGAGAAAAGTAGGACATT | 22 | 45.487 | 29.829 |
| bmo-miR-965-3p | TAAGCGTATAGCTTTTCCCCTT | 22 | 488.987 | 407.132 |
| bmo-miR-993b-3p | AAAGCTCGTCTCTACAGGTATAT | 23 | 5.686 | 5.643 |
| bmo-miR-3372-5p | TTTATTATGTTCTTGGCGTCAGAGCCAAA | 29 | 5.686 | 0.806 |
| bmo-miR-3304a | TAAACAGCTTGGAATATTTACAG | 23 | 0 | 3.225 |
| bmo-miR-2827 | CAGACTATCAGTACGTACGCTG | 22 | 0 | 9.674 |
| bmo-miR-8-5p | CATCTTACCGGGCAGCATTAGA | 22 | 1066.106 | 2533.895 |
| bmo-miR-33-5p | GTGCATTGTAGTTGCATTGCA | 21 | 159.205 | 108.031 |
| bmo-miR-3384-3p | TGTCTGTTTGTGCGCTGGTAGAAGA | 25 | 0 | 3.225 |
| bmo-miR-279c-3p | TGACTAGATCCATACTCGTCTG | 22 | 9836.606 | 6780.97 |
| bmo-let-7-3p | CTGTATAGCCTGCTAACTTTCC | 22 | 1481.177 | 865.055 |
| bmo-miR-3387-3p | TTCAGGTTCGTGCATAATGTGCT | 23 | 2.843 | 3.225 |
| bmo-miR-79-3p | TTCATAAAGCTAGATTACCAAAGCAT | 26 | 2334.062 | 1332.653 |
| bmo-miR-2807d | CAGCTGCATACAAAATTGGGC | 21 | 0 | 1.612 |
| bmo-miR-2822 | AGGGATATTTTACTCCGCTT | 20 | 0 | 0.806 |
| bmo-miR-282-5p | ACCTAGCCTCTCCTTGGCTTTGTCTGT | 27 | 250.18 | 1036.776 |
| bmo-miR-932 | TCAATTCCGTAGTGCATTGCAG | 22 | 0 | 1.612 |
| bmo-miR-277-3p | TAAATGCACTATCTGGTACGACA | 23 | 537.317 | 2553.243 |
| bmo-miR-79-5p | CTTTGGCGATTTAGCTCCGTGA | 22 | 88.131 | 104 |
| bmo-miR-2799 | AGAGGTTTATGAACATGATGAG | 22 | 2.843 | 0 |
| bmo-miR-2849 | AACTTTACGATTATGGACTCAG | 22 | 54.016 | 66.109 |
| bmo-miR-285 | TAGCACCATTCGAATTCAGTGC | 22 | 14.215 | 27.411 |
| bmo-miR-2761-3p | TGTGTGGAACCGTCGTCGATGGA | 23 | 8.529 | 6.45 |
| bmo-miR-3333 | CCGTTCGAGAAGCAAGACAAAGTG | 24 | 39.801 | 128.186 |
| bmo-miR-190-3p | CCCGGGAATCAAACATATTACTCT | 24 | 130.776 | 233.799 |
| bmo-miR-306a-5p | TCAGGTACTAGGTGACTCTGA | 21 | 48435.334 | 26119.35 |
| bmo-miR-2762 | GTACGTCGGGAAATGTACGGTA | 22 | 0 | 0 |
| bmo-miR-2778b-3p | CAGAGTAGGCAAAAAAACAATT | 22 | 2.843 | 2.419 |
| bmo-miR-2733i-3p | TCACTGGGAATGTAATGACTAT | 22 | 88.131 | 327.318 |
| bmo-miR-3001 | TAAGTTGAAAGAATTGTAGATTTTGA | 26 | 963.76 | 479.691 |
| bmo-miR-970-5p | AGCCTTGCGTGTGCTCTTATTGGTA | 25 | 17.058 | 52.403 |
| bmo-miR-2a-3p | TATCACAGCCAGCTTTGATGAGC | 23 | 1330.5 | 2380.716 |
| bmo-miR-2859 | CAACTATTGATCGGGCAGGACAA | 23 | 0 | 0 |
| bmo-miR-306b | TGAGGCACGAGGCGGCTCTGA | 21 | 2.843 | 4.837 |
| bmo-miR-2792-3p | TAGATTAGATAGCGATTCCATT | 22 | 0 | 5.643 |
| bmo-miR-3404 | TGAATCTTTTCTGCAATAGTGTGAC | 25 | 2.843 | 3.225 |
| bmo-miR-1a-5p | CCGTGCTTCCTTACTTCCCAT | 21 | 5.686 | 10.481 |
| bmo-miR-3213-5p | TACTACTTTACAGGGAATGCTCAGC | 25 | 2.843 | 2.419 |
| bmo-miR-2847 | TATTGTGTTTGTCAGTGCGGTATA | 24 | 0 | 1.612 |
| bmo-miR-2842 | TGAAGATCCTCGTACTGGTGGCGC | 24 | 5.686 | 4.031 |
| bmo-miR-750-3p | CCAGATCTATCTTTCCAGCT | 20 | 123497.735 | 22534.167 |
| bmo-miR-2794 | ACGGCGACTTTTCGTAGCGA | 20 | 0 | 0 |
| bmo-miR-2833b | AGAGATTGGAAATGTACTTTT | 21 | 2.843 | 9.674 |
| bmo-miR-317-5p | CGGGTGCCACGCTGTGCTCTCT | 22 | 8.529 | 20.961 |
| bmo-miR-993b-5p | TACCCTGTAGATCCGGGCTTTCG | 23 | 287.138 | 143.504 |
| bmo-miR-2733f | TCACTGGGTATGTAATGACAGT | 22 | 45.487 | 95.132 |
| bmo-miR-927-5p | TTTAGAATTCCTACGCTTTACC | 22 | 116.561 | 318.45 |
| bmo-miR-3374-5p | TGAAGATGCACCAGATGTTGAGAGGCC | 27 | 5.686 | 4.031 |
| bmo-miR-3388-5p | ACAAGTCAAACATGCCTTGGCCCATTCA | 28 | 8.529 | 28.217 |
| bmo-miR-3363 | TCATACGTATTTGTGTACTGCGTTT | 25 | 8.529 | 8.062 |
| bmo-miR-1175-3p | TGAGATTCAACTCCTCCAACTTAA | 24 | 5694.428 | 328.124 |
| bmo-miR-9b-3p | ACGGAGCTAAATCGCCAAAGCG | 22 | 2.843 | 1.612 |
| bmo-miR-34-5p | TGGCAGTGTGGTTAGCTGGTTG | 22 | 235.965 | 176.558 |
| bmo-miR-2780a-3p | AATCGTTTGAATGCTCGTCTAA | 22 | 17.058 | 16.124 |
| bmo-miR-2778a-2-5p | GTTTTTTTGTATATCCTGC | 19 | 0 | 0 |
| bmo-miR-2807a | TTCCAGCTGCATACAAATTTCA | 22 | 2.843 | 1.612 |
| bmo-miR-2770-3p | TTATCCCCGTGTACTGTTAG | 20 | 2.843 | 0 |
| bmo-miR-124 | TAAGGCACGCGGTGAATGCCAAG | 23 | 0 | 1.612 |
| bmo-miR-3332 | TCCTCGCCATGCCACCGCCGCTTCA | 25 | 0 | 4.031 |
| bmo-miR-2768-3p | ATTGGTTAAGATATTGCATCGT | 22 | 2.843 | 10.481 |
| bmo-miR-7-5p | TGGAAGACTAGTGATTTTGTTGT | 23 | 102.346 | 50.791 |
| bmo-miR-283-3p | CAGGCTATCAGCTGGTATACAG | 22 | 25.587 | 3.225 |
| bmo-miR-3338-3p | ATGTACTTACTTTGTTTGTTCT | 22 | 0 | 349.086 |
| bmo-miR-13b-5p | TCGTAAAAATGGCTGTGTCGTG | 22 | 54.016 | 26.605 |
| bmo-miR-14-3p | TCAGTCTTTTTCTCTCTCCTA | 21 | 8565.807 | 12973.411 |
| bmo-miR-3373-5p | TAGCCGCAGAACTGTTCGGGCAT | 23 | 2.843 | 3.225 |
| bmo-miR-279d-5p | GATGAGTGACCGTTTAGTTCAA | 22 | 102.346 | 78.202 |
| bmo-miR-3343 | TCTCCATCGTTCCTGCACCGTAGC | 24 | 31.272 | 20.155 |
| bmo-miR-2777-5p | AGGGACCTTGCCTGGGTTAT | 20 | 99.503 | 87.07 |
| bmo-miR-2733h | TCACTGGGTGTATGATGATTG | 21 | 0 | 0.806 |
| bmo-miR-2778a-3p | CAGAGTACGCAAAAAAACAATT | 22 | 5509.637 | 3883.478 |
| bmo-miR-92b | AATTGCACCAATCCCGGCCTGC | 22 | 1299.228 | 1154.482 |
| bmo-miR-2817 | AACAAGACTGCGTAGACCGAG | 21 | 5.686 | 12.093 |
| bmo-miR-2836 | CATCCCGCTGTAGACGACGC | 20 | 14.215 | 5.643 |
| bmo-miR-2737 | TTTTATACTGTCCAGATTTGT | 21 | 0 | 0.806 |
| bmo-miR-3391-5p | AGGTCGTGGTATTTCGTACCTTCATGG | 27 | 0 | 0.806 |
| bmo-miR-2763-5p | TGCAAAGTAGTGAACATAATTTAT | 24 | 0 | 0.806 |
| bmo-bantam-5p | CTGGTTTTCATAATGATTTGACA | 23 | 122.247 | 102.388 |
| bmo-miR-279d-3p | TGACTAGATTTTCACTTATCCT | 22 | 33609.351 | 20296.149 |
| bmo-miR-2778d-3p | CAGAGTACGCAAAAAACAATT | 21 | 14.215 | 8.062 |
| bmo-miR-3323 | CAATACGAATAATGTTAATGCCT | 23 | 0 | 5.643 |
| bmo-miR-1923 | TAATCGCGTACCGTTGCATAGCCGTGGC | 28 | 0 | 0.806 |
| bmo-miR-2845 | CCGTTGCCAGCTGCTGTGCGTA | 22 | 25.587 | 29.829 |
| bmo-miR-3297-5p | GCACATCTAGTCAAAAATATTGGAG | 25 | 0 | 0.806 |
| bmo-miR-2766-3p | TCAGTCTTGTCGAATGGTG | 19 | 14587.175 | 16234.501 |
| bmo-miR-92a | TATTGCACCAGTCCCGGCCTAT | 22 | 187.635 | 195.907 |
| bmo-miR-3339 | TAGACTTTTTAGATGTTGGTCAA | 23 | 11.372 | 0.806 |
| bmo-miR-3378-5p | TTCCATGTTGATCGGGGCTGCTTC | 24 | 0 | 0.806 |
| bmo-miR-14-5p | CGGGGAGAGAAATCGACGAGGCT | 23 | 1822.331 | 3175.632 |
| bmo-miR-2843-5p | TCTAAGGAAATTAGGTCGGATACA | 24 | 51.173 | 66.109 |
| bmo-miR-2759 | TGAAAGATCATAGAATGCGAAA | 22 | 11.372 | 8.062 |
| bmo-miR-100 | AACCCGTAGATCCGAACTTGTG | 22 | 8847.259 | 14897.817 |
| bmo-miR-71-5p | TGAAAGACATGGGTAGTGA | 19 | 28.429 | 16.93 |
| bmo-miR-3308-3p | CTAGGCAATGGGACAAAATCA | 21 | 0 | 1.612 |
| bmo-miR-2810 | AGATTTTCGAGAACAGCTAATT | 22 | 2.843 | 0.806 |
| bmo-miR-275-3p | TCAGGTACCTGAAGTAGCGCGCG | 23 | 3761.222 | 10421.78 |
| bmo-miR-3420 | TACTGTTATTGCCTGGCCTCGCTCA | 25 | 0 | 2.419 |
| bmo-miR-316-3p | ACGGCAAAGTGAAAAGGTCTCC | 22 | 76.76 | 41.923 |
| bmo-miR-3362 | TGATTTGAAGTTTATGAATGTTGTA | 25 | 0 | 2.419 |
| bmo-miR-274-3p | TCGTTTTGGCGATCGCAAAATG | 22 | 690.837 | 593.365 |
| bmo-miR-2832 | TTGGATAGTGCGTTTTGGATGTC | 23 | 0 | 2.419 |
| bmo-miR-3389-3p | CCCTGCGGAACACCGCGCTCG | 21 | 0 | 0 |
| bmo-miR-2839-5p | TCAAACAGAGCGCCAGCGCTAT | 22 | 2.843 | 0 |
| bmo-miR-3403 | TGAATCTAGTCTGAAGTCTGATTTG | 25 | 5.686 | 5.643 |
| bmo-miR-3000 | CTGCGCTTAGATGAAGACACTA | 22 | 48.33 | 94.326 |
| bmo-miR-2777-3p | AGAGCGCAGGGGAGGTGCTTAG | 22 | 0 | 13.705 |
| bmo-miR-2851 | TGTGATGACTGTATTAGAGAGA | 22 | 0 | 0 |
| bmo-miR-2808d | CCGGCGAGAAGCTCGGTGTGCT | 22 | 0 | 0.806 |
| bmo-miR-3383-5p | GTGCCTGAGGGCTTCGTCCATGTT | 24 | 0 | 0 |
| bmo-miR-277-5p | TCGTGCCAGGAGTGCGTTTGC | 21 | 2.843 | 31.442 |
| bmo-miR-8-3p | TAATACTGTCAGGTAAAGATGTC | 23 | 61936.502 | 54298.548 |
| bmo-miR-3388-3p | TCGCATTGGGACCTGGATGACTGA | 24 | 0 | 5.643 |
| bmo-miR-2733i-5p | AGTTGTTACTTCCTCAGATGA | 21 | 2.843 | 3.225 |
| bmo-miR-2795 | CAAGTTTGGTGATACGCGGGCGC | 23 | 102.346 | 89.488 |
| bmo-miR-2808e | TGAGAAGATCCGGTGAGTAACT | 22 | 5.686 | 4.031 |
| bmo-miR-2766-5p | CCGCCCTTCGTCTTGACTGGCG | 22 | 11502.575 | 6908.35 |
| bmo-miR-375-5p | ACCCGAGCGGTCTGAGCAAACT | 22 | 525.946 | 1351.195 |
| bmo-miR-3414-3p | TGATTTTTTGTTCGATGTCAACCTA | 25 | 0 | 3.225 |
| bmo-miR-2828 | ATATTCGATATGTGAACGGTT | 21 | 2.843 | 1.612 |
| bmo-miR-3302 | TACGAATTTCAGTCTGATTGCAA | 23 | 0 | 0.806 |
| bmo-miR-2785 | TGCAGTGCGGAACGAGGCTAA | 21 | 0 | 0 |
| bmo-miR-184-3p | ACTGGACGGAGAACTGATAAGGGC | 24 | 33268.198 | 34298.275 |
| bmo-miR-2820 | CAGCAAGATGGAACGTGAATA | 21 | 0 | 0 |
| bmo-miR-308-5p | CGTGGTATTATTCTTGTGAATGT | 23 | 855.728 | 520.807 |
| bmo-miR-31-5p | GGCAAGAAGTCGGCATAGCTG | 21 | 52671.329 | 53116.655 |
| bmo-miR-9c-5p | TCTTTGGTATCCTAGCTG | 18 | 26072.692 | 11179.611 |
| bmo-miR-252-5p | CTAAGTACTAGTGCCGCAGGAG | 22 | 673.779 | 1437.459 |
| bmo-miR-278-3p | TCGGTGGGATCTTCGTCCGTTT | 22 | 3260.863 | 3802.857 |
| bmo-miR-12 | TGAGTATTACTTCAGGTACTGGT | 23 | 5768.345 | 1260.901 |
| bmo-miR-3400 | GCAGCTTGCAAGTCATTTTCTTAG | 24 | 0 | 4.031 |
| bmo-miR-2763-3p | TATTATGCTCATTTCTTTGGAT | 22 | 5.686 | 882.792 |
| bmo-miR-965-5p | CGGGAGAAGCTATAGCGCTATATG | 24 | 1540.879 | 577.241 |
| bmo-miR-3267a | TGGCTTTGAGCTCGTCCACCCATT | 24 | 19.901 | 27.411 |
| bmo-miR-2998 | AAGAACAGGATGAGGTAGATAAA | 23 | 51.173 | 58.047 |
| bmo-miR-3220 | TTCTTTCCGCGCGTGACGGTTTGGA | 25 | 0 | 3.225 |
| bmo-let-7-5p | TGAGGTAGTAGGTTGTATAGT | 21 | 3729.95 | 2911.197 |
| bmo-miR-2733g | TCACTGGGTGCATGAAGATTG | 21 | 0 | 4.837 |
| bmo-miR-2821 | GTCACGTAGGGCACCGGTGA | 20 | 0 | 0 |
| bmo-miR-263a-3p | CGTGATCTCTTAGTGGCATCAC | 22 | 36.958 | 44.341 |
| bmo-miR-1b-5p | CCATACTTCTTTACATTCCATA | 22 | 2.843 | 3.225 |
| bmo-miR-2797d | ATAAGTAGACATTGTCCGGCTT | 22 | 0 | 5.643 |
| bmo-miR-137 | TTATTGCTTGAGAATACACGTA | 22 | 5.686 | 10.481 |
| bmo-miR-2745 | TAAATTCGGTCTTTCGGGC | 19 | 1412.946 | 1649.49 |
| bmo-miR-34-3p | AGCCACTAACGACACTGCTCCT | 22 | 39.801 | 99.163 |
| bmo-miR-9b-5p | GCTTTGGTAATCTAGCTTTATGA | 23 | 2.843 | 2.419 |
| bmo-miR-210 | TTGTGCGTGTGACAGCGGCT | 20 | 2.843 | 0 |
| bmo-miR-1000 | ATATTGTCCTGTCACAGCAGT | 21 | 2.843 | 4.837 |
| bmo-miR-750-5p | AGTTGGACAGGGGATCTTGACA | 22 | 906.901 | 323.287 |
| bmo-miR-3329 | GCATACAATAATTTATGACAGAT | 23 | 0 | 7.256 |
| bmo-miR-2841 | ATCATAGTTAAGAGCTCAAAA | 21 | 5.686 | 1.612 |
