# Supplementary material for: Genome-Wide Analysis of Differentially Expressed microRNA in Bombyx mori Infected with Nucleopolyhedrosis Virus
Source: PLoS One. 2016 Nov 2;11(11):e0165865. doi: 10.1371/journal.pone.0165865 (PMC5091789; doi:10.1371/journal.pone.0165865)
Supplement: S3 Table — (DOCX) [file pone.0165865.s003.docx]

S3 Table. Novel miRNAs identified in two small libraries

| miRNA_Name | Sequence | Length | Control(TPM) | Infection(TPM) | miRNA with same seed sequence |
| --- | --- | --- | --- | --- | --- |
| novel-22398 | CCUGAGAUGAUAGGGGAUCGCA | 22 | 4244.55 | 0 | dme-miR-285-5p |
| novel-22184 | GUAAGCAGAUUAUGUUCGACU | 21 | 2122.275 | 1014.591 | hsa-miR-6792-5p |
| novel-40665 | CGGACACGGUGCGGGCCGCGCUG | 23 | 0 | 811.673 | pma-miR-4622 |
| novel-40241 | UUGACAAUGUUAAUGAAUCGCU | 22 | 0 | 811.673 | ame-miR-6058 |
| novel-23505 | GAGUGUUCGACGGGGUAAC | 19 | 36078.674 | 5478.791 | hsa-miR-4255 |
| novel-23887 | UAAUUCGGGGACUUUAGAAAU | 21 | 4951.975 | 405.836 | lmi-miR-10-3p |
| novel-39855 | UGACUAGACCCUAAACAGGGUAAU | 24 | 7074.25 | 2840.855 | cel-miR-44-3p |
| novel-25024 | ACGUGUGCGCGCAGUGCGGACA | 22 | 1414.85 | 1826.264 | mmu-miR-696 |
| novel-25022 | AAUUUUCUCGCCGAACCCUUCG | 22 | 0 | 4870.037 | nve-miR-9467 |
| novel-9699 | CUAGGUACUGGCUUGUGG | 18 | 21930.174 | 19277.229 | dps-miR-2558-5p |
| novel-9949 | UCUUCGUCUCAUACAAAGUAUG | 22 | 707.425 | 0 | hco-miR-5972 |
| novel-26898 | UACGGGUGACUGGAUGCUUCCA | 22 | 707.425 | 811.673 | mml-miR-552 |
| novel-41619 | GAAAUUUUGAUAUUACGUAAUG | 22 | 707.425 | 0 | cfa-miR-8838 |
| novel-41422 | GAAAUUUUGAUAUUACGUAAUG | 22 | 707.425 | 0 | cfa-miR-8838 |
| novel-30549 | ACCCAAUGUUCGUUAGUGAUAU | 22 | 56593.998 | 168422.103 | gga-miR-6676-3p |
| novel-29973 | AUGGAUUUGGAACAGCUUCCCU | 22 | 8489.1 | 7305.055 | rno-miR-3595 |
| novel-42257 | UGGGGCUCGUACCUGACUUGG | 21 | 0 | 2232.1 | hsa-miR-4283 |
| novel-32464 | GGGGUGUUGUCGCUGUCUGAGUGGU | 25 | 343101.112 | 278809.602 | hsa-miR-609 |
| novel-33021 | UUGGGCUUGACCCAGCAAGGACU | 23 | 1414.85 | 1217.509 | bta-miR-2308 |
| novel-32892 | UCACUGGGAAUGUAAUAACUAU | 22 | 18393.049 | 66151.332 | dme-miR-3-3p |
| novel-32888 | UCACUGGGUAUGUAAUAGCUAU | 22 | 11318.8 | 71224.287 | dme-miR-3-3p |
| novel-32633 | UCAACUGAGAGUAUGAUGACU | 21 | 1414.85 | 1014.591 | sme-miR-7a-3p |
| novel-33506 | UACUGGCCUGCUAAGUCCCAAG | 22 | 2829.7 | 6899.219 | mmu-miR-193a-3p |
| novel-33828 | UGUUCCAAGAUAUAAUGUAUACG | 23 | 0 | 608.755 | bfl-miR-4906 |
| novel-42780 | UACUUUGAUCAAUAUGUUCAGU | 22 | 3537.125 | 608.755 | sme-miR-2169-5p |
| novel-34405 | CAAAAUCACUAGUCUUCCAUACA | 23 | 0 | 405.836 | sko-miR-7-3p |
| novel-34663 | UGACAUCACGUGCGCGCGCUGCU | 23 | 5659.4 | 8928.401 | hsa-miR-3913-3p |
| novel-7977 | GCGGCUGUUAACUUUAGGCAGC | 22 | 0 | 2637.937 | dvi-miR-313-5p |
| novel-12574 | UGGCAUGUACCACGUCAAGGUCA | 23 | 5659.4 | 3246.691 | bbe-miR-2068-3p |
| novel-36790 | GUAAGUAGAAAAUUCUGUGUCU | 22 | 336026.862 | 240052.227 | cel-miR-251 |
| novel-36575 | CCCCUGCACUCUUGCACCCG | 20 | 1414.85 | 4261.282 | bta-miR-2331-3p |
| novel-14868 | AUCUGUAUAGCAAUGCACGCCA | 22 | 0 | 0 | mml-miR-7207-3p |
| novel-38271 | CAUUGAGAAGAUCCGGCGGGA | 21 | 19807.899 | 25973.529 | hsa-miR-4461 |
| novel-44289 | UGCCUUGUAGUCAUAUUGCACG | 22 | 1414.85 | 1014.591 | hsa-miR-4312 |
| novel-44259 | AAUAUACUCUAUAGGAUAACCU | 22 | 5659.4 | 11160.501 | sme-miR-9b-3p |
| novel-44000 | AUCUUUUUGCAUGCCGGCAGAUG | 23 | 707.425 | 811.673 | cin-miR-4000b-2-3p |
| novel-1721 | CUAGGUACUGGCUUGUGG | 18 | 21930.174 | 19277.229 | dps-miR-2558-5p |
| novel-23837 | UGCUGAAAAGGAGUCGCCGC | 20 | 1414.85 | 608.755 |  |
| novel-22398* | UGCGACUCUUACCAUCUCUGGCA | 23 | 0 | 0 |  |
| novel-40244* | GUGGGGCAAAUUGCGAAAGCUG | 22 | 1414.85 | 608.755 |  |
| novel-40244 | UUUCGCAAUUUGCCGCACCC | 20 | 2122.275 | 1826.264 |  |
| novel-25022* | ACGGGUUCGACGAGAACG | 18 | 0 | 202.918 |  |
| novel-9949* | AUACUUUGUAUGAGAUGAAGAU | 22 | 0 | 0 |  |
| novel-25024* | UCCGGCCUGCGCGCGCACGCC | 21 | 0 | 202.918 |  |
| novel-39950* | UUUAUGACUUAGGCUUAGUUG | 21 | 1414.85 | 2840.855 |  |
| novel-39950 | UCUAGGCCCGCCUCAUGAACU | 21 | 9903.95 | 14001.356 |  |
| novel-10803* | UGGUGGCUUCUCUGAGUACUUG | 22 | 0 | 0 |  |
| novel-10803 | AGUGCAGAGAGAAUCCAUCAUG | 22 | 0 | 1420.427 |  |
| novel-30843* | AUACGCACCGAGUACUCGGCCG | 22 | 707.425 | 0 |  |
| novel-30843 | CCCGUUUACUCGGUACGUAUGAU | 23 | 1414.85 | 2232.1 |  |
| novel-11075* | UGGUGGCUUCUCUGAGUACUUG | 22 | 0 | 0 |  |
| novel-11075 | AGUGCAGAGAGAAUCCAUCAUG | 22 | 0 | 1420.427 |  |
| novel-32293* | CUAGAACAUUCUCAAUCGAA | 20 | 0 | 0 |  |
| novel-32293 | CUUCGAUUGCUAGAACGUUCUAGAA | 25 | 707.425 | 608.755 |  |
| novel-33021* | UUGCUGGGCCAAGCCCAAA | 19 | 707.425 | 0 |  |
| novel-32892* | AGUUGUUACUUCCUCAGGCGAC | 22 | 0 | 1826.264 |  |
| novel-32888* | AGUUGUUACUUCCUCAGUCGACA | 23 | 707.425 | 2029.182 |  |
| novel-33506* | AGGGUCUUGGCGGUCUGGUCGGU | 23 | 0 | 202.918 |  |
| novel-34405* | UAGGCAGAUUAGUGAUUUCUUGUU | 24 | 0 | 405.836 |  |
| novel-34663* | CGGCACUGCACGUGAUCUCACU | 22 | 6366.825 | 5275.873 |  |
| novel-12574* | UACCUUGACGCAGUACAUGCCA | 22 | 0 | 1420.427 |  |
| novel-8123 | GCUAAGUGCAUUAGUGGGUCUAU | 23 | 707.425 | 1014.591 |  |
| novel-13009* | UGAAGGAUCAAUGUAGAAGAUC | 22 | 0 | 202.918 |  |
| novel-13009 | UUUUCUACAUUGGUCCUUCACU | 22 | 1414.85 | 0 |  |
| novel-36790* | ACUCAAAAUUAUCUUCUUUCAG | 22 | 5659.4 | 9334.237 |  |
| novel-14868* | CAUGUCAUUGAUAUACAGGAUGA | 23 | 0 | 0 |  |
| novel-17979* | CGUACCCUGGAACGCACAAU | 20 | 707.425 | 0 |  |
| novel-17979 | CUUUGCGUUCCAGGGUACGCCU | 22 | 0 | 811.673 |  |
| novel-39227 | UAUCUAAUCGCUAGGAGCCU | 20 | 0 | 3652.528 |  |
| novel-44289* | UGCAAUGCAACUACAAUGCACU | 22 | 707.425 | 0 |  |
| novel-1_44259* | GAUAACCUAUAGAAUAUAUUUUAA | 24 | 0 | 202.918 |  |
| novel-1_44115* | ACUCAGCAAUGCCAGUGUCGGCU | 23 | 6366.825 | 4667.119 |  |
| novel-1_44115 | ACGACAUGGUGUUGCUGAGCCU | 22 | 5659.4 | 9131.319 |  |
