# Supplementary material for: Genome-Wide Analysis of Differentially Expressed microRNA in Bombyx mori Infected with Nucleopolyhedrosis Virus
Source: PLoS One. 2016 Nov 2;11(11):e0165865. doi: 10.1371/journal.pone.0165865 (PMC5091789; doi:10.1371/journal.pone.0165865)
Supplement: S4 Table — (DOCX) [file pone.0165865.s004.docx]

S4 Table. Primer sequences used in this study

| MicroRNA | Primer | Primer sequence(5'-3') |
| --- | --- | --- |
| Novel-32888 | RT | CTCAACTGGTGTCGTGGAGTCGGCAATTCAGTTGAGAGACACAG |
|  | Forward | ACACTCCAGCTGGGGTAAGTAGAAAATT |
| Novel-32892 | RT | CTCAACTGGTGTCGTGGAGTCGGCAATTCAGTTGAGATAGTTAT |
|  | Forward | ACACTCCAGCTGGGTCACTGGGAATGTA |
| Novel-30549 | RT | CTCAACTGGTGTCGTGGAGTCGGCAATTCAGTTGAGATATCACT |
|  | Forward | ACACTCCAGCTGGGACCCAATGTTCGTT |
| Novel-32464 | RT | CTCAACTGGTGTCGTGGAGTCGGCAATTCAGTTGAGACCACTCA |
|  | Forward | ACACTCCAGCTGGGGGGGTGTTGTCGCTGTC |
| Novel-36790 | RT | CTCAACTGGTGTCGTGGAGTCGGCAATTCAGTTGAGAGACACAG |
|  | Forward | ACACTCCAGCTGGGGTAAGTAGAAAATT |
| Novel-23505 | RT | CTCAACTGGTGTCGTGGAGTCGGCAATTCAGTTGAGGTTACCCC |
|  | Forward | ACACTCCAGCTGGGGAGTGTTCGAC |
| bmo-miR-3338-3p | RT | CTCAACTGGTGTCGTGGAGTCGGCAATTCAGTTGAGAGAACAAA |
|  | Forward | ACACTCCAGCTGGGATGTACTTACTTTG |
| bmo-miR-2764 | RT | CTCAACTGGTGTCGTGGAGTCGGCAATTCAGTTGAGCCAGTAAC |
|  | Forward | ACACTCCAGCTGGGTTCGTAGATATTGTA |
| bmo-miR-2763-3p | RT | CTCAACTGGTGTCGTGGAGTCGGCAATTCAGTTGAGATCCAAAG |
|  | Forward | ACACTCCAGCTGGGTATTATGCTCATTT |
| bmo-miR-282-5p | RT | CTCAACTGGTGTCGTGGAGTCGGCAATTCAGTTGAGACAGACAA |
|  | Forward | ACACTCCAGCTGGGACCTAGCCTCTCCTTGGCT |
| bmo-miR-277-5p | RT | CTCAACTGGTGTCGTGGAGTCGGCAATTCAGTTGAGGCAAACGC |
|  | Forward | ACACTCCAGCTGGGTCGTGCCAGGAGT |
| bmo-miR-10-5p | RT | CTCAACTGGTGTCGTGGAGTCGGCAATTCAGTTGAGACAAATTC |
|  | Forward | ACACTCCAGCTGGGACCCTGTAGATCC |
| bmo-miR-1175-5p | RT | CTCAACTGGTGTCGTGGAGTCGGCAATTCAGTTGAGTGAAGAGA |
|  | Forward | ACACTCCAGCTGGGAAGTGGAGGTGTGA |
| bmo-miR-1175-3p | RT | CTCAACTGGTGTCGTGGAGTCGGCAATTCAGTTGAGTTAAGTTG |
|  | Forward | ACACTCCAGCTGGGTGAGATTCAACTCCTC |
| bmo-miR-750-3p | RT | CTCAACTGGTGTCGTGGAGTCGGCAATTCAGTTGAGAGCTGGAA |
|  | Forward | ACACTCCAGCTGGGCCAGATCTATCT |
| U6 | Forward | CGTATACTAAAATTGGAACGATACAG |
|  | Reward | ATTTTGCGTGTCATCCTTGC |
| Universal reverse | | TGGTGTCGTGGAGTCG |
| bmo-miR-277-5p mimic | | 5’- UCGUGCCAGGAGUGCGUUUGCGU-3’  5’- GCAAACGCACUCCUGGCACGAUU-3’ |
| Negative control | | 5’-UUCUCCGAACGUGUCACGUTT-3’  5’-ACGUGACACGUUCGGAGAATT-3’ |
